# Comparing Agreement Indices to Assess Inter-Observer Reliability in the Case of Dichotomous and Trichotomous Animal-Based Welfare Indicators with Three Raters

**DOI:** 10.3390/ani16040546

**Published:** 2026-02-10

**Authors:** Benedetta Torsiello, Mauro Giammarino, Piero Quatto, Monica Battini, Silvana Mattiello, Luca Battaglini, Manuela Renna

**Affiliations:** 1 Department of Agricultural, Forest and Food Sciences, University of Turin, Largo Paolo Braccini 2, 10095 Grugliasco, TO, Italy; luca.battaglini@unito.it; 2 Department of Prevention, Asl TO3, Veterinary Service, Animal Health Unit, Via Torino 62, 10045 Piossasco, TO, Italy; mauro.giammarino@aslto3.piemonte.it; 3 Department of Economics, Quantitative Methods and Business Strategies, University of Milan-Bicocca, Piazza dell’Ateneo Nuovo 1, 20126 Milan, MI, Italy; piero.quatto@unimib.it; 4 Department of Agricultural and Environmental Sciences—Production, Landscape, Agroenergy, University of Milan, Via Celoria 2, 20133 Milan, MI, Italy; monica.battini@unimi.it (M.B.); silvana.mattiello@unimi.it (S.M.); 5 Department of Veterinary Sciences, University of Turin, Largo Paolo Braccini 2, 10095 Grugliasco, TO, Italy; manuela.renna@unito.it

**Keywords:** agreement index, animal-based measure, bootstrap method, dichotomous variable, inter-observer reliability, trichotomous variable

## Abstract

This study deals with the evaluation of inter-observer reliability (IOR) among three raters in the case of dichotomous and trichotomous individual animal-based welfare indicators. The performance of the most documented agreement indices proposed in the literature was compared, using udder asymmetry (UA) as a dichotomous indicator and body condition score (BCS) as a trichotomous indicator, both obtained from the AWIN Goat protocol. Nine dairy goat farms, exploiting three alpine pastures (AP1 to AP3), were used for data collection. Krippendorff’s *α*, the agreement indices belonging to the Kappa statistic and their weighted forms were in some cases affected by the paradox behaviour. This phenomenon was observed for both UA and BCS [e.g., P_0(BCS-AP2)_ = 80%; Fleiss’ *K* = 0.22]. In the case of UA, Gwet’s *γ*(*AC*_1_), followed by *BP* coefficient and Quatto’s *S*, gave the best agreement results [e.g., P_0(UA-AP1)_ = 86%; *γ*(*AC*_1_) = 0.84]. In the case of BCS, the best agreement results were obtained with Gwet’s *γ*(*AC*_2_), followed by the weighted forms of *BP* and *S*. When the evaluation is performed by three raters, *γ*(*AC*_1_), *BP* and *S* are suggested to evaluate IOR in the case of both dichotomous and trichotomous indicators, while the related weighted forms are suitable for trichotomous indicators only.

## Simple Summary

Nowadays, the evaluation of inter-observer reliability is of outmost importance for ensuring the introduction of individual animal-based welfare indicators within animal welfare protocols. The present study focuses on the evaluation of inter-observer reliability of dichotomous and trichotomous individual animal-based welfare indicators (assessed through two/three levels scoring system), which is guaranteed calculating the concordance among three raters during the evaluation process through some statistical indices proposed in the current literature, defined as agreement indices. In this regard, the performance of the most popular agreement indices is compared to understand which ones are the most suitable to assess the inter-observer reliability. The most exploited agreement indices (e.g., the indices belonging to the Kappa statistic) are shown to be inappropriate to evaluate the inter-observer reliability in the presence of three raters. On the contrary, some less known agreement indices, such as Gwet’s *γ*(*AC*_1_), Gwet’s *γ*(*AC*_2_), Quatto’s *S*, Quatto’s weighted *S*, Brennan and Prediger’s *BP* coefficient and Brennan and Prediger’s weighted *BP*, were able to confer more reliable agreement results.

## 1. Introduction

Animal-based welfare indicators confer accurate information regarding the real welfare status of a subject [1], measuring the reactions of the animal to both the resources present inside the environment where it lives (resource-based welfare indicators) and its manipulation by humans (management-based welfare indicators) [2].

Together with validity and feasibility, reliability is one of the most important features for an animal-based welfare indicator to be included into animal welfare protocols [3]. A relevant type of reliability is the inter-observer reliability (IOR) [4], which is linked to the level of agreement between two or more raters, when they classify independent sample units inside a predetermined category [5] at the same time and without influencing each other [6].

A proper IOR evaluation is guaranteed developing some statistical indices proposed in the literature, defined as agreement indices, which confer a value of the concordance among the raters during the evaluation process [7]. Subsequently, the obtained concordance value is compared to the concordance rate (P_0_), which is given by the ratio between the number of times that the raters agree out of the total number of observations [8]. Thus, the lower is the concordance among the raters, the lower will be the IOR of an indicator.

Most of the animal-based welfare indicators contained inside currently available animal welfare protocols are dichotomous and trichotomous variables which are evaluated using two- and three-level scoring, respectively. Giammarino et al. [9] and Torsiello et al. [6] identified the most suitable agreement indices to assess the IOR for dichotomous and trichotomous variables in the presence of two raters. However, welfare assessment can be performed by different assessors for different purposes (e.g., self-assessment, official controls, and certification procedures); therefore, it is also fundamental to assess which are the most suitable indices to evaluate the agreement among multiple raters for the above-mentioned variables.

In this regard, as already reported for two raters [6,9], also in the presence of multiple raters, a critical point is represented by the implementation of the rate of agreement that occurs by chance (P_e_), which must be removed from the P_0_ [10]. Gwet [11] stated that adjusting the concordance rate for the chance agreement becomes crucial in the presence of multiple raters, due to the constraints imposed by the experimental design. Specifically, Gwet [11] made the example of three raters, who can classify a subject into two categories only. In this case, the three raters will not have the possibility to completely disagree, due to the low presence of categories. Consequently, two out of three raters will necessarily agree on this classification, with the possibility that some of the agreements will be due to chance.

The P_e_ is calculated in different ways, depending on the formula of the implemented agreement index [7]. For example, Fleiss [12] defined the chance agreement as the probability to assign a subject into the same category by a single pair of raters. Krippendorff [13] followed the same approach, even if, differently from Fleiss [12], he proposed a statistic which is applicable in the presence of both two and multiple raters [6]. Conger [14] criticised this assumption, stating that the P_e_ can be implemented averaging the probabilities of assignments of a subject into the same category not only by a single pair but by all the involved pairs of raters.

However, as already pointed out for two raters [6,9], also in the presence of three raters, the agreement indices belonging to the Kappa statistic (Fleiss’ *K* [12], Light’s *K* [15], Hubert’s *K* [16], and Conger’s *K* [14]) can be affected by the paradox behaviour [17]. Indeed, when the P_e_ assumes high values, the agreement values obtained implementing the Kappa statistic can sometimes result very low if compared to the P_0_ [17]. On the contrary, some agreement indices which calculate the P_e_ taking into consideration the number of categories that characterises the variable under analysis are free from the paradox and are suitable to assess the IOR properly [6].

Based on the above-reported considerations, the aim of this study is to identify the most suitable agreement indices for properly assessing the IOR of dichotomous and trichotomous animal-based welfare indicators in the presence of three raters. For this purpose, we selected two indicators from a modified version of the original AWIN welfare assessment protocol for goats [18], namely the udder asymmetry (UA) and body condition score (BCS), which are evaluated using two- and three-level scoring, respectively.

## 2. Materials and Methods

### 2.1. Dichotomous and Trichotomous Animal-Based Welfare Indicators

A modified version of the AWIN protocol developed for the welfare assessment of dairy goats kept under semi-extensive farming systems [18] was applied by three raters in nine dairy goat farms, exploiting three alpine pastures (APs) which were breeding a total of 160 goats (AP1: n = 44; AP2: n = 70; and AP3: n = 46), in north-west Italy, between June and August 2021. Two raters were enrolled in the second year of the MSc in Animal Science, while the third observer was enrolled in the first year of the MSc in Forestry and Environmental Sciences, at the University of Turin (Italy). The raters had no previous experience with dairy goats. Before data collection, the raters received both a theoretical and a practical training by one of the authors of the original AWIN protocol developed for the welfare assessment of dairy goats kept under intensive and semi-intensive farming systems [19]. In addition, as training material, they received both the original AWIN protocol [19] and the adapted AWIN protocol to be applied in semi-extensive farming conditions [18]. The theoretical training was based on these protocols and on additional training material developed within the AWIN project and made available to the raters, consisting of Power Point presentations divided into the following different sections: (1) Definition of the indicator; (2) How to assess it; (3) How to score it; (4) Examples (four photos for UA and 15 for BCS, plus three detailed drawings for BCS); and (5) Self-assessment (six questions for each indicator, where raters can test their knowledge and ability to correctly assess the indicator with immediate feedback on the correct/incorrect answer). After all the raters correctly assessed the indicators using the theoretical training material, a practical training was carried out with an expert trainer (one of the authors of the AWIN protocols) on a farm raising about 80 lactating goats. The indicators were further described, including methods and scoring systems, and discussed with practical examples. Raters were then asked to simultaneously assess UA and BCS and then their assessment was discussed to agree on the scoring and to clarify uncertainties. Only when all the raters assessed goats giving the same scores, the training was considered satisfactory (one full day was required).

The UA and BCS were chosen as dichotomous and trichotomous categorical animal-based welfare indicators, respectively. The UA was confirmed when one half of the udder was at least 25% longer than the other, excluding the teats [19]. According to the original AWIN protocol [19], for UA each goat was assigned to one of two mutually exclusive and exhaustive categories (absence of asymmetry = 0; presence of asymmetry = 1). This binary classification had previously been confirmed as a good predictor of the somatic cell count and the microorganism present in the udder [20]. For BCS each goat was assigned to one of three mutually exclusive and exhaustive categories (very thin goat = −1; normal goat = 0; and very fat goat = 1). Both indicators were recorded for all the goats in the three APs.

### 2.2. Agreement Indices and Confidence Intervals

The simplest measure for assessing the reliability is the P_0_. Fleiss [12] defined P_0_ as the ratio between the number of times that the pairs of raters agree in assigning the scores to each subject involved in the evaluation and the total number of subjects. However, this assumption was already previously criticised by Cohen [21], as P_0_ does not consider the possibility of agreement occurring by chance [22]. For this reason, to estimate the IOR properly, it is fundamental to implement agreement indices which also consider the P_e_.

The most documented agreement indices implemented to assess the IOR for dichotomous and trichotomous variables, in the presence of multiple raters, are reported in Table 1.

In particular, Krippendorff’s *α* [13], Fleiss’ *K* [12], Light’s *K* [15], Hubert’s *K* [16], Conger’s *K* [14], *BP* coefficient [24], Quatto’s *S* [25], Gwet’s *γ*(*AC*_1_) [27] and Andrès and Hernàndez’s multi-raters Δ [28] are the most documented in the current literature to evaluate the IOR among three raters for both dichotomous and trichotomous indicators. In the case of trichotomous indicators, in addition to the above-mentioned agreement indices, Krippendorff’s weighted *α* (α*; [29]), Gwet’s *γ*(*AC*_2_) [11], Fleiss’ weighted *K* (K*; [11]), Conger’s weighted *K* (K*; [11]), weighted *BP* coefficient (BP*; [11]), Quatto’s weighted *S* (S*; [26]) and Hubert’s weighted *K* (K*; [30]) are also available.

The closed formulas of the above-mentioned agreement indices are reported in the Supplementary Materials.

For each agreement index, the calculation of the confidence intervals is fundamental to gather information regarding the dispersion of the values assumed by the index itself inside the sample. For this purpose, the calculation of the variance estimates is a prerequisite to guarantee the implementation of the confidence intervals.

### 2.3. Statistical Analyses

Differently from the approach applied in the case of two raters by Giammarino et al. [9] and Torsiello et al. [6], in the current study the manual implementation of the agreement indices based on closed formulas was not performed, because the complexity of the calculation increases as the number of raters increases.

R Commander (version R × 64 4.2.2) was used to implement both the values and the confidence intervals of all the considered agreement indices. The Bootstrap *t*-method [31] was implemented to calculate values and confidence intervals of all the agreement indices, while some packages and R functions were specifically developed to calculate the values only (i.e., Krippendorff’s *α*, Fleiss’ *K*, Light’s *K*, and Andrès and Hernàndez’s multi-raters Δ) or both the values and the confidence intervals (i.e., Krippendorff’s *α*, Fleiss’ *K*, Conger’s *K*, *BP* coefficient, Quatto’s *S*, Krippendorff’s α*, Gwet’s *γ*(*AC*_1_), Gwet’s *γ*(*AC*_2_), Fleiss’s K*, Conger’s K*, BP* coefficient, and Quatto’s S*) of the agreement indices.

All the indices and confidence intervals were calculated separately for each AP.

All the exploited R packages (version R × 64 4.2.2) and functions are summarised in Table 1.

## 3. Results

### 3.1. Dichotomous Animal-Based Welfare Indicators

#### 3.1.1. Agreement Indices for Udder Asymmetry

The values of the agreement indices obtained for UA in each AP are reported in Table 2.

Two different concordance rates (P_01_; P_02_) were obtained, based on the agreement index under analysis. The first one (P_01_) was the same for all the agreement indices, except for Hubert’s *K* and Andrès and Hernàndez’s multi-raters Δ, for which a different concordance rate (P_02_) was obtained.

In AP1, despite the fact that P_01_ and P_02_ were equal to 86% and 80%, respectively, Krippendorff’s *α* and all the indices belonging to the Kappa statistic (i.e., Fleiss’ *K*, Light’s *K*, Hubert’s *K*, and Conger’s *K*) resulted in negative values (−0.07 to −0.03). In both AP2 and AP3, the results obtained for the above-mentioned agreement indices were above zero; however, their values (AP2: 0.51 and 0.52; AP3: 0.68 and 0.69) were low if compared to their respective concordance rates (AP2: P_01_ = 92% and P_02_ = 89%; AP3: P_01_ = 94% and P_02_ = 91%).

The *BP* coefficient, Quatto’s *S* and Gwet’s *γ*(*AC*_1_) conferred agreement results close to P_01_ in all the considered cases. Gwet’s *γ*(*AC*_1_) values (0.84, 0.91 and 0.93 in AP1, AP2 and AP3, respectively) were closer to those of the concordance rate (P_01_) if compared to *BP* coefficient and Quatto’s *S* ones (0.73, 0.85 and 0.88, respectively). Andrès and Hernàndez’s multi-raters Δ gave agreement results in line with the respective concordance rate (P_02_) in two out of three APs (AP2: P_02_ = 89%, Δ = 0.79; AP3: P_02_ = 91%, Δ = 0.86); however, in AP1 this index exceeded the concordance rate (P_02_ = 80%, Δ = 0.86).

#### 3.1.2. Confidence Intervals for Udder Asymmetry

The values of the confidence intervals obtained for UA in each AP are shown in Table 3.

The confidence intervals implemented using the Bootstrap *t*-method and, when available, using specific R functions, were very close to each other in all the APs, resulting identical in some cases.

Wide confidence intervals were obtained in AP2 and AP3 for Krippendorff’s *α* and for the indices belonging to the Kappa statistic. For the same agreement indices, the confidence intervals in AP1 were narrow, but characterised by negative values. Gwet’s *γ*(*AC*_1_), *BP* coefficient, Quatto’s *S* and Andrès and Hernàndez’s multi-raters Δ conferred tight confidence intervals in all the studied cases.

### 3.2. Trichotomous Animal-Based Welfare Indicators

#### 3.2.1. Agreement Indices for Body Condition Score

The values of the agreement indices obtained for BCS are reported in Table 4.

As already observed for dichotomous indicators, also for trichotomous ones, the obtained concordance rates were not the same for all the implemented agreement indices (P_01_; P_02_; and P_03_). In this regard, P_01_ was the concordance rate obtained for Krippendorff’s *α*, almost all the agreement indices belonging to the Kappa statistic (except for Hubert’s *K*), *BP* coefficient, Quatto’s *S* and Gwet’s *γ*(*AC*_1_). P_02_ was the concordance rate obtained for Hubert’s *K* and Andrès and Hernàndez’s multi-raters Δ, while P_03_ was obtained for all the weighted indices (i.e., Krippendorff’s α*, Gwet’s *γ*(*AC*_2_), Fleiss’s K*, Conger’s K*, BP* coefficient, Quatto’s S* and Hubert’s K*).

For BCS the agreement results obtained for Krippendorff’s *α*, the indices belonging to the Kappa statistic and the related weighted forms were very low in all the APs if compared to their respective concordance rates. In AP3, despite high concordance rates (P_01_ = 80%; P_02_ = 70%; and P_03_ = 90%), such values were close to zero (0.02 to 0.07). On the other hand, *BP* coefficient, Quatto’s *S* and Gwet’s *γ*(*AC*_1_) showed agreement results in line with the related concordance rate (P_01_). Moreover, as already pointed out for dichotomous variables, Gwet’s *γ*(*AC*_1_) values were closer to the concordance rate if compared to those obtained for *BP* coefficient and Quatto’s *S*. The agreement values obtained for Andrès and Hernàndez’s multi-raters Δ were close to the related concordance rate in AP1 and AP2 (P_02_ = 77% and 70%; Δ = 0.75 and 0.65, respectively), while in AP3 the result for this index was quite low if compared to P_02_ (0.47 and 70%, respectively). Gwet’s *γ*(*AC*_2_), BP* coefficient and Quatto’s S* showed agreement values in line with the observed concordance rate (P_03_) in all the considered cases. The results given by Gwet’s *γ*(*AC*_2_) were closer to P_03_ if compared to those obtained for BP* coefficient and Quatto’s S*.

#### 3.2.2. Confidence Intervals for Body Condition Score

As already observed for dichotomous indicators (Table 3), also for the trichotomous ones, the results of the confidence intervals obtained exploiting the Bootstrap t-method were very close to those obtained implementing the R functions (Table 5).

In particular, it is known that the R functions “concordance” and “wlin.conc”, used for the implementation of Quatto’s *S* and Quatto’s S*, respectively, were developed starting from the Bootstrap method.

Krippendorff’s *α*, the indices belonging to the Kappa statistic and their relative weighted forms conferred wide confidence intervals in AP1 and AP2, while in AP3 the confidence intervals obtained for the above-mentioned indices were tighter. On the other hand, *BP* coefficient, Quatto’s *S* and Gwet’s *γ*(*AC*_1_) were characterised by narrow confidence intervals in all the APs. Such trends resemble what was already observed for dichotomous indicators (Table 3).

The confidence intervals for Andrès and Hernàndez’s multi-raters Δ were tight in AP1 and AP2, while they were wide in AP3. Gwet’s *γ*(*AC*_2_), BP* coefficient and Quatto’s S* showed narrow confidence intervals in all the considered APs.

## 4. Discussion

The UA is treated as a categorical variable, identifying the absence or the presence of the welfare problem only. On the other hand, the BCS can be considered both as a categorical and ordinal variable, as it is possible to identify a pre-ordered scale, based on the accumulation of body fat reserves [6].

Most of the agreement indices implemented in the current study are suitable to assess the IOR in the presence of both two and multiple raters (i.e., Krippendorff’s *α*; *BP* coefficient; Quatto’s *S*; Krippendorff’s α*; Gwet’s *γ*(*AC*_1_); Gwet’s *γ*(*AC*_2_); BP* coefficient; and Quatto’s S*).

Based on the type of concordance matrix (i.e., agreement matrix) developed for the calculation of the agreement indices considered in the current study, three different P_0_ are obtained (Table 2 and Table 4; Supplementary Materials). Fleiss [12] proposed a concordant matrix where the sum of the squared probabilities through which each subject is attributed to a specific category allows to obtain the P_0_. The same approach is used when implementing Krippendorff’s *α*, Light’s *K*, Conger’s *K*, *BP* coefficient, Quatto’s *S* and Gwet’s *γ*(*AC*_1_). Andrès and Hernàndez [28] proposed an alternative type of concordance matrix, valid for the implementation of both Andrès and Hernàndez’s multi-raters Δ and Hubert’s *K*, based on the contingencies table implemented by Dillon and Mulani [32]. Finally, in the case of weighted agreement indices, which are specifically developed to assess the reliability of variables evaluated through an ordered scale, Krippendorff [29], Gwet [11], Marasini et al. [26] and Andrès and Hernàndez [30] proposed a concordance matrix for the calculation of Krippendorff’s α*, Gwet’s *γ*(*AC*_2_), Fleiss’ K*, Conger’s K*, BP* coefficient, Quatto’s S* and Hubert’s K*, where the level of disagreement among the raters is also considered.

In this regard, the unweighted indices only allow to quantify and to verify the presence or absence of the agreement among the raters when they classify a subject within a predetermined category. On the contrary, the weighted forms of these indices also measure the degree of the disagreement present among the raters during this classification [33]. For example, concerning the ordinal variable analysed in the current study (BCS), a higher disagreement among the raters when they classify the subjects within the categories −1 or 1 (very thin goat; very fat goat) could be considered more serious if compared to disagreement observed for the categories 0 and 1 (normal goat; very fat goat), as the difference present between these two latter categories is lower if compared to the previous ones, and the possibility of error during this last classification is higher and more acceptable. Consequently, the disagreement among the raters for the categories −1 and 1 “weighs more” during the evaluation if compared to the disagreement obtained for the categories 0 and 1. The seriousness of this kind of disagreement is evaluated implementing a matrix where specific weights, proposed in the current literature [11], are exploited.

Our results show that the agreement indices belonging to the Kappa statistic can be affected by the paradox behaviour for both dichotomous (Table 2) and trichotomous (Table 4) indicators, conferring, in some cases, very low agreement values despite high P_0_ [17]. The paradox has been widely analysed in the published literature [17,34,35], especially when discussing the evaluation of agreement among two raters using Cohen’s *K* [21]. However, it is known that the paradox behaviour also affects the Kappa indices in the presence of multiple raters. In this regard, Falotico and Quatto [36] stated that the statistic proposed by Fleiss [12] should be invariant to the permutation, that is, the different combinations of assignments when couples of raters identify a subject within a specific category. However, this statement is not verified for Fleiss’ *K*, which gets worse when the marginal distributions within the concordance matrix are not fixed. This means that the raters are unaware regarding the exact number of times each subject is attributed to each of the categories characterising the variable. Thus, each subject involved in the evaluation process can be attributed with no limits to a specific category, producing constant assignments, but resulting in higher variations and unbalanced marginal distributions [37]. Consequently, this factor conduces to the obtainment of lower agreement values if compared to P_0_ (Table 2 and Table 4). Furthermore, Fleiss’ *K* is an extension of Scott’s *π* [38,39], which is sometimes affected by the paradox behaviour when assessing the IOR in the presence of two raters [6,9]. Conger [14] tried to improve the Kappa statistic proposed by Fleiss [12], but our results clearly show that Conger’s *K* can also sometimes confer low agreement values if compared to P_0_, therefore being susceptible to paradox behaviours (Table 2 and Table 4). Similarly, Light’s *K* [15] and Hubert’s *K* [16], being generalisations of Cohen’s *K* in the presence of multiple raters [39], are also affected by the above-mentioned problem (Table 2 and Table 4). Krippendorff’s *α* suffers from the paradox behaviour too, when the evaluation is performed by two [6,9] or multiple raters (current study). Furthermore, the weighted forms of the above-mentioned agreement indices are affected by the same problem, even if they were able to confer slightly higher agreement results if compared to the respective unweighted forms (Table 4). The same trend was observed for Cohen’s weighted *K* [40] when assessing the IOR of trichotomous indicators in the presence of two raters [6].

For all these indices, the occurrence of paradox behaviour is also highlighted when observing the obtained confidence intervals. The complexity of the manual implementation of closed formulas of variance estimates increases as the number of the raters and categories increase. While it can be quite easily applied in the presence of two raters [6,9], it becomes challenging already in the presence of three raters. For this reason, in the current study, the closed formulas of variance estimates were not implemented manually to calculate the confidence intervals. The Bootstrap *t*-method proposed by Efron [31] was already previously recommended to be used for confidence intervals calculation [6,9], as it is easier than the manual implementation of closed formulas. Moreover, through a resampling technique, bootstrapping also allows obtaining a more accurate implementation of the confidence intervals if compared to closed formulas, which are based on approximate calculation of the variance estimates, resulting worse in terms of accuracy and flexibility [41].

For the agreement indices affected by the paradox behaviour, the confidence intervals result is wide, showing a high dispersion of the values obtained within the sample (Table 3 and Table 5). Tight confidence intervals are sometimes found for agreement indices affected by the above-mentioned problem; in particular, this occurs when the indices, and their confidence intervals, result in negative values (Table 3 and Table 5). Such results confirm what was previously observed when assessing the IOR for dichotomous and trichotomous indicators in the presence of two raters [6,9].

Despite the occurrence of the paradox behaviour, the Kappa statistic, and especially Fleiss’ *K*, has been frequently exploited in the published literature to assess the IOR of animal-based welfare indicators in the presence of multiple raters. For example, Fleiss’ *K* was developed to assess the IOR of indicators used to evaluate the presence of respiratory diseases in pre-weaned dairy calves [42], the rumen fill and tail length in ewes kept outdoors [43], the presence of severe lameness in horses [44], the kneel fracture in laying hens [45], and ten different indicators of lamb welfare (i.e., demeanour, response to stimulation, shivering, standing ability, posture, abdominal fill, body condition, lameness, eye condition and salivation) [46]. Examples of the paradox behaviour are also highlighted in the literature when the IOR of animal-based welfare indicators was assessed for several species and in the presence of many raters (P_0_ = 86%; Fleiss’ *K* = 0.13 [42]; P_0_ = 73%; Fleiss’ *K* = 0.14 [43]; P_0_ = 61.9%; Fleiss’ *K* = 0.23 [44]; P_0_ = 94%; Fleiss’ *K* = 0.13 [47]; P_0_ = 81%; and Fleiss’ *K* = 0.43 [48]). Similarly, Torsiello et al. [6] recently highlighted examples of the paradox behaviour in the published literature when evaluating the IOR of trichotomous and four-level animal-based welfare indicators in the presence of two raters.

Alternative agreement indices have been proposed more recently to overcome the paradox that may affect older indices. Andrès and Marzo’s Δ [49] was developed to solve this problem and confers good agreement results when assessing the IOR for dichotomous indicators in the presence of two raters [9]. The same trend is visible in the current study for UA when implementing Andrès and Hernàndez’s multi-raters Δ [28] even if, in AP1, this index overestimates the P_0_ (Table 2). This phenomenon can be explained considering that multi-raters Δ is not developed starting from the implementation of the real agreement present among the raters but starting from the calculation of the estimated agreement when the raters classify the subjects involved within each predetermined category [28]. Consequently, based on estimations, the agreement values obtained for multi-raters Δ cannot be necessarily correct and could result in values which can exceed the P_0_. Another explanation of the overestimated value obtained for multi-raters Δ relies on the unbalance of the agreement present among the raters towards a specific category. Specifically, in the current study, the agreement was very high for the category 0 (Δ = 0.88) but resulted in a negative value for the category 1 (Δ = −0.02), producing an overall agreement which exceeded the P_0_ (Δ = 0.86) (Table 2). As reported by Andrès and Hernàndez [28], this means that the raters should try to improve the criteria used when they classify a subject within a specific category, in order to homogenise the degree of agreement for each of the considered categories (consistency), consequently obtaining more reliable agreement values.

The confidence intervals obtained for Δ are tighter in the presence of dichotomous indicators, resulting in a lower dispersion of the values within the sample, if compared to the indices belonging to the Kappa statistic (Table 3). Concerning the BCS, Andrès and Hernàndez’s multi-raters Δ [28] confers better agreement results in the presence of three raters (Table 4) if compared to the ones obtained with Andres and Marzo’s Δ in the presence of two raters [6]. Despite this, in AP3, the agreement value obtained for this index is worse when comparing it to P_0_ (Table 4), also resulting in wider confidence intervals (Table 5).

The agreement indices which implement the P_e_ considering the total number of categories characterising the variable are paradox-free and conferred in all the APs the best agreement results for both UA and BCS (Table 2 and Table 4). This occurs because these indices are not influenced by the unbalanced values assumed by the marginal distributions within the concordance matrix when the assignments of the subjects to a specific category are higher, as instead observed for the Kappa statistic [17]. For example, Quatto [25] calculated the P_e_ as the inverse of the total number of categories, and this principle is valid in the presence of both two and multiple raters. Furthermore, the *S*-statistic follows the same statistical approach of *BP* coefficient [24], which is a generalisation of Holley and Guilford’s *G* [50] and Bennett, Alpert and Goldstein’s *S* [51] in the presence of two raters for variables characterised by three categories or more [11]. Following the same statistical approach, Quatto’s *S* and *BP* coefficient showed identical agreement values in all considered cases (Table 2 and Table 4).

In the presence of multiple raters, Gwet [11] defined the P_e_ as the probability that pairs of raters, casually selected from a group of *n* raters, agree on assigning a subject into a predetermined category. Thus, after calculating the P_e_ for each pair of raters, the total chance agreement is given averaging the values of all the above-mentioned P_e_.

Gwet’s *γ*(*AC*_1_), *BP* coefficient, and Quatto’s *S* gave the best results for both dichotomous and trichotomous categorical indicators. Moreover, their weighted forms (Gwet’s *γ*(*AC*_2_), BP* coefficient and Quatto’s S* (BP* coefficient and Quatto’s S* follow the same statistical approach)) conferred the best agreement values for trichotomous ordinal indicators. When using weighted agreement indices, a crucial point is always represented by the choice of the weights used for the implementation of the index itself. For the current study, as described in Torsiello et al. [6], the linear weights proposed by Cicchetti and Allison [52] were exploited (the same weights are also used for the development of the weighted forms of the Kappa indices, and for Krippendorff’s α*) as they are less sensitive to the total number of categories of the analysed variable [26]. Furthermore, Gwet’s *γ*(*AC*_1_), *BP* coefficient, and Quatto’s *S*, as well as their relative weighted forms, also conferred the narrowest confidence intervals (Table 3 and Table 5), pointing out a low dispersion of the values obtained within the sample. This trend has also been observed when assessing the IOR for both dichotomous and trichotomous indicators in the presence of two raters [6,9].

## 5. Conclusions

From the results obtained in this study, it is evident that Krippendorff’s *α*, the Kappa indices and their weighted forms can suffer from the paradox behaviour when evaluating the IOR of dichotomous and trichotomous animal-based welfare indicators in the presence of multiple raters. For this reason, Gwet’s *γ*(*AC*_1_), *BP* coefficient, Quatto’s *S*, and their weighted forms (Gwet’s *γ*(*AC*_2_), BP* coefficient, Quatto’s S*), being paradox-free, should be preferred when assessing the IOR of dichotomous and trichotomous animal-based welfare indicators in the presence of three raters. Gwet’s *γ*(*AC*_1_), *BP* coefficient and Quatto’s *S* consider the total number of categories characterising the variable when implementing the P_e_; for this reason, they can be considered suitable to assess the IOR of categorical variables, characterised by any number of categories, and in the presence of both two and many raters. The same approach is followed by their weighted forms, which are recommended to assess the IOR in the presence of ordinal variables and in the presence of any number of raters.

The confidence intervals also give relevant information regarding the accuracy and the goodness of the implemented agreement indices. In this regard, the best agreement indices are those characterised by tighter confidence intervals. The Bootstrap *t*-method and R functions (when available) can be considered valid methods to calculate the agreement values and their relative confidence intervals for each considered agreement index, avoiding the use of cumbersome closed formulas, and demonstrating usefulness in the presence of both two [6,9] and many raters.

Finally, it has to be highlighted that UA and BCS, used in the present study, are only examples of dichotomous and trichotomous variables, and that the considerations drawn from our results can be generalised and applied to other dichotomous and trichotomous animal-based welfare indicators, particularly to those for which information on reliability is not available yet (which is a common issue, especially for pasture-based systems; [53]), also for different farm species. As stated above, reliability is one of the most important features for animal-based welfare indicators: the availability of appropriate tools to evaluate IOR is therefore of fundamental importance for selecting the most appropriate indicators, especially when different raters are called to assess welfare, above all for certification purposes, to ensure a fair assessment.

## Figures and Tables

**Table 1:** Agreement indices implemented for each animal-based welfare indicator.

VARIABLES	AGREEMENT INDICES	REFERENCES FOR THE AGREEMENT INDEX	CONFIDENCE INTERVALS	REFERENCES FOR THE CONFIDENCE INTERVALS	R PACKAGES (VERSION R × 64 4.2.2)	R FUNCTIONS
**UA** **and** **BCS**	Krippendorff’s *α*	Krippendorff [13]	Bootstrap	Krippendorff [13]	library (irr) library (irrCAC) library (boot)	boot_result var (boot) boot.ci kripp.alpha krippen.alpha.raw
	Fleiss’ *K*	Fleiss [12]	Bootstrap	Fleiss [12]	library (irr) library (raters) library (irrCAC) library (boot)	boot_result var (boot) boot.ci kappam.fleiss fleiss.kappa.raw concordance
	Light’s *K*	Light [15]	Bootstrap	Light [15]	library(irr) library(boot)	boot_result var (boot) boot.ci kappam.light
	Hubert’s *K*	Hubert [16]	Bootstrap	Hubert [16] Andrès and Hernàndez [23]	library (boot)	boot_result var (boot) boot.ci
	Conger’s *K*	Conger [14]	Bootstrap	Conger [14]	library (irrCAC) library (boot)	boot_result var (boot) boot.ci conger.kappa.raw
	*BP* coefficient	Brennan and Prediger [24]	Bootstrap	Gwet [11]	library (irrCAC) library (boot)	boot_result var (boot) boot.ci bp.coeff.raw
	Quatto’s *S*	Quatto [25]	Bootstrap	Quatto [25] Marasini et al. [26]	library (raters) library (boot)	boot_result var (boot) boot.ci concordance
	Gwet’s *γ*(*AC*_1_)	Gwet [27]	Bootstrap	Gwet [27]	library (irrCAC) library (boot)	boot_result var (boot) boot.ci gwet.ac1.raw
	Andrès and Hernàndez’s multi-raters Δ	Andrès and Hernàndez [28]	Bootstrap	Andrès and Hernàndez [28]	library (DeltaMAN) library (boot)	boot_result var (boot) boot.ci multiDelta
**BCS**	Krippendorff’s weighted *α*	Krippendorff [29]	Bootstrap	Gwet [11]	library (irrCAC) library (boot)	boot_result var (boot) boot.ci krippen.alpha.raw
	Gwet’s *γ*(*AC*_2_)	Gwet [11]	Bootstrap	Gwet [11]	library (irrCAC) library (boot)	boot_result var (boot) boot.ci gwet.ac1.raw
	Fleiss’ weighted *K*	Gwet [11]	Bootstrap	Gwet [11]	library (irrCAC) library (boot)	boot_result var (boot) boot.ci fleiss.kappa.raw
	Conger’s weighted *K*	Gwet [11]	Bootstrap	Gwet [11]	library(irrCAC) library(boot)	boot_result var (boot) boot.ci conger.kappa.raw
	Weighted *BP* coefficient	Gwet [11]	Bootstrap	Gwet [11]	library (irrCAC) library (boot)	boot_result var (boot) boot.ci bp.coeff.raw
	Quatto’s weighted *S*	Marasini et al. [26]	Bootstrap	Marasini et al. [26]	library (raters) library (boot)	boot_result var (boot) boot.ci wlin.conc
	Hubert’s weighted *K*	Andrès and Hernàndez [30]	Bootstrap	Andrès and Hernàndez [30]	library (boot)	boot_result var (boot) boot.ci

**Table 2:** Values of the concordance rate and of the agreement indices obtained for udder asymmetry for the three alpine pastures.

	Concordance Rate	Agreement Indices	Agreement Values
**AP1** **(n = 44)**	P_01_ = 86% P_02_ = 80%	*α*	−0.07
		Fleiss’ *K*	−0.07
		Light’s *K*	−0.03
		Hubert’s *K*	−0.04
		Conger’s *K*	−0.04
		*BP*	0.73
		*S*	0.73
		*γ*(*AC*_1_)	0.84
		Δ	0.86
**AP2** **(n = 70)**	P_01_ = 92% P_02_ = 89%	*α*	0.52
		Fleiss’ *K*	0.51
		Light’s *K*	0.51
		Hubert’s *K*	0.51
		Conger’s *K*	0.51
		*BP*	0.85
		*S*	0.85
		*γ*(*AC*_1_)	0.91
		Δ	0.79
**AP3** **(n = 46)**	P_01_ = 94% P_02_ = 91%	*α*	0.68
		Fleiss’ *K*	0.68
		Light’s *K*	0.69
		Hubert’s *K*	0.68
		Conger’s *K*	0.68
		*BP*	0.88
		*S*	0.88
		*γ*(*AC*_1_)	0.93
		Δ	0.86

Abbreviations: n = sample size; P_01_ = concordance rate for Krippendorff’s *α*, Fleiss’ *K*, Light’s *K*, Conger’s *K*, Brennan and Prediger’s coefficient, Quatto’s *S* and Gwet’s *γ*(*AC*_1_); P_02_ = concordance rate for Hubert’s *K* and Andrès and Hernàndez’s multi-raters Δ; *α* = Krippendorff’s *α*; *BP* = Brennan and Prediger’s coefficient; *S* = Quatto’s *S*; *γ*(*AC*_1_) = Gwet’s *γ*(*AC*_1_); Δ = Andrés and Hernàndez’s multi-raters Δ; AP1 = alpine pasture 1; AP2 = alpine pasture 2; AP3 = alpine pasture 3.

**Table 3:** Values of the confidence intervals for the agreement indices obtained for udder asymmetry implemented using the Bootstrap *t*-method and R functions in the three alpine pastures.

		Confidence Intervals	
	Agreement Indices	By Bootstrap *t*-Method	By R Functions
**AP1** **(n = 44)**	*α*	−0.11; −0.02	krippen.alpha.raw: −0.11; −0.02
	Fleiss’ *K*	−0.12; −0.03	fleiss.kappa.raw: −0.12; 0.03
	Light’s *K*	−0.05; 0.00	N.A.
	Hubert’s *K*	−0.08; 0.00	N.A.
	Conger’s *K*	−0.08; 0.00	conger.kappa.raw: −0.08; 0.01
	*BP*	0.57; 0.89	bp.coeff.raw: 0.56; 0.89
	*S*	0.57; 0.89	concordance: 0.58; 0.88
	*γ*(*AC*_1_)	0.74; 0.95	gwet.ac1.raw: 0.74; 0.95
	Δ	0.80; 0.94	N.A.
**AP2** **(n = 70)**	*α*	0.18; 0.92	krippen.alpha.raw: 0.20; 0.84
	Fleiss’ *K*	0.20; 0.88	fleiss.kappa.raw: 0.19; 0.84
	Light’s *K*	0.19; 0.89	N.A.
	Hubert’s *K*	0.20; 0.89	N.A.
	Conger’s *K*	0.19; 0.90	conger.kappa.raw: 0.19; 0.84
	*BP*	0.75; 0.94	bp.coeff.raw: 0.75; 0.95
	*S*	0.75; 0.95	concordance: 0.73; 0.94
	*γ*(*AC*_1_)	0.85; 0.98	gwet.ac1.raw: 0.84; 0.98
	Δ	0.67; 0.92	N.A.
**AP3** **(n = 46)**	*α*	0.37; 1.05	krippen.alpha.raw: 0.38; 0.99
	Fleiss’ *K*	0.36; 1.10	fleiss.kappa.raw: 0.37; 0.99
	Light’s *K*	0.35; 1.08	N.A.
	Hubert’s *K*	0.34; 1.09	N.A.
	Conger’s *K*	0.36; 1.07	conger.kappa.raw: 0.38; 0.99
	*BP*	0.77; 1.00	bp.coeff.raw: 0.77; 1.00
	*S*	0.78; 0.99	concordance: 0.77; 0.97
	*γ*(*AC*_1_)	0.86; 1.00	gwet.ac1.raw: 0.86; 1.00
	Δ	0.75; 0.96	N.A.

Abbreviations: n = sample size; *α* = Krippendorff’s *α*; *BP* = Brennan and Prediger’s coefficient; *S* = Quatto’s *S*; *γ*(*AC*_1_) = Gwet’s *γ*(*AC*_1_); Δ = Andrés and Hernàndez’s multi-raters Δ; AP1 = alpine pasture 1; AP2 = alpine pasture 2; AP3 = alpine pasture 3; N.A. = not available (i.e., no R function available to calculate confidence intervals).

**Table 4:** Values of the concordance rate and of the agreement indices obtained for body condition score for the three alpine pastures.

	Concordance Rate	Agreement Indices	Agreement Values
**AP1** **(n = 44)**	P_01_ = 85% P_02_ = 77% P_03_ = 92%	*α*	0.35
		Fleiss’ *K*	0.35
		Light’s *K*	0.36
		Hubert’s *K*	0.33
		Conger’s *K*	0.35
		*BP*	0.77
		*S*	0.77
		*γ*(*AC*_1_)	0.83
		Δ	0.75
		α*	0.37
		*γ*(*AC*_2_)	0.91
		Fleiss’ K*	0.37
		Conger’s K*	0.37
		BP*	0.83
		S*	0.83
		Hubert’s K*	0.37
**AP2** **(n = 70)**	P_01_ = 80% P_02_ = 70% P_03_ = 90%	*α*	0.23
		Fleiss’ *K*	0.22
		Light’s *K*	0.24
		Hubert’s *K*	0.21
		Conger’s *K*	0.23
		*BP*	0.70
		*S*	0.70
		*γ*(*AC*_1_)	0.77
		Δ	0.65
		α*	0.24
		*γ*(*AC*_2_)	0.87
		Fleiss’ K*	0.24
		Conger’s K*	0.24
		BP*	0.78
		S*	0.78
		Hubert’s K*	0.24
**AP3** **(n = 46)**	P_01_ = 80% P_02_ = 70% P_03_ = 90%	*α*	0.04
		Fleiss’ *K*	0.04
		Light’s *K*	0.04
		Hubert’s *K*	0.02
		Conger’s *K*	0.04
		*BP*	0.70
		*S*	0.70
		*γ*(*AC*_1_)	0.77
		Δ	0.47
		α*	0.07
		*γ*(*AC*_2_)	0.88
		Fleiss’ K*	0.06
		Conger’s K*	0.07
		BP*	0.77
		S*	0.77
		Hubert’s K*	0.07

Abbreviations: n = sample size; P_01_ = concordance rate for Krippendorff’s *α*, Fleiss’ *K*, Light’s *K*, Conger’s *K*, Brennan and Prediger’s coefficient; Quatto’s *S* and Gwet’s *γ*(*AC*_1_); P02 = concordance rate for Hubert’s *K* and Andrès and Hernàndez’s multi-raters Δ; P_03_ = concordance rate for Krippendorff’s weighted *α*, Gwet’s *γ*(*AC*_2_), Fleiss’ weighted *K*, Conger’s weighted *K*, Brennan and Prediger’s weighted coefficient, Quatto’s weighted *S* and Hubert’s weighted *K*; *α* = Krippendorff’s *α*; *BP* = Brennan and Prediger’s coefficient; *S* = Quatto’s *S*; *γ*(*AC*_1_) = Gwet’s *γ*(*AC*_1_); Δ = Andrés and Hernàndez’s multi-raters Δ; α* = Krippendorff’s weighted *α*; *γ*(*AC*_2_) = Gwet’s *γ*(*AC*_2_); Fleiss’ K* = Fleiss’ weighted *K*; Conger’s K* = Conger’s weighted *K*; BP* = Brennan and Prediger’s weighted coefficient; S* = Quatto’s weighted *S*; Hubert’s K* = Hubert’s weighted *K*; AP1 = alpine pasture 1; AP2 = alpine pasture 2; AP3 = alpine pasture 3.

**Table 5:** Values of the confidence intervals for the agreement indices obtained for body condition score implemented using the Bootstrap *t*-method and R functions in three alpine pastures.

		Confidence Intervals	
	Agreement Indices	By Bootstrap *t*-Method	By R Functions
**AP1** **(n = 44)**	*α*	0.11; 0.64	krippen.alpha.raw: 0.09; 0.62
	Fleiss’ *K*	0.11; 0.62	fleiss.kappa.raw: 0.08; 0.61
	Light’s *K*	0.13; 0.63	N.A.
	Hubert’s *K*	0.07; 0.61	N.A.
	Conger’s *K*	0.11; 0.62	conger.kappa.raw: 0.09; 0.61
	*BP* coefficient	0.65; 0.90	bp.coeff.raw: 0.64; 0.90
	*S*	0.65; 0.90	concordance: 0.64; 0.89
	*γ*(*AC*_1_)	0.73; 0.93	gwet.ac1.raw: 0.72; 0.94
	Δ	0.58; 0.89	N.A.
	α*	0.13; 0.65	krippen.alpha.raw: 0.11; 0.64
	*γ*(*AC*_2_)	0.85; 0.97	gwet.ac1.raw: 0.84; 0.97
	Fleiss’ K*	0.12; 0.64	fleiss.kappa.raw: 0.10; 0.63
	Conger’s K*	0.13; 0.65	conger.kappa.raw: 0.11; 0.63
	BP*	0.74; 0.93	bp.coeff.raw: 0.73; 0.93
	S*	0.73; 0.92	wlin.conc: 0.73; 0.91
	Hubert’s K*	0.14; 0.65	N.A.
**AP2** **(n = 70)**	*α*	0.06; 0.40	krippen.alpha.raw: 0.06; 0.40
	Fleiss’ *K*	0.06; 0.40	fleiss.kappa.raw: 0.05; 0.40
	Light’s *K*	0.07; 0.41	N.A.
	Hubert’s *K*	0.05; 0.39	N.A.
	Conger’s *K*	0.07; 0.40	conger.kappa.raw: 0.06; 0.40
	*BP* coefficient	0.59; 0.80	bp.coeff.raw: 0.59; 0.81
	*S*	0.59; 0.81	concordance: 0.59; 0.80
	*γ*(*AC*_1_)	0.69; 0.86	gwet.ac1.raw: 0.68; 0.86
	Δ	0.44; 0.88	N.A.
	α*	0.09; 0.41	krippen.alpha.raw: 0.08; 0.41
	*γ*(*AC*_2_)	0.82; 0.93	gwet.ac1.raw: 0.82; 0.93
	Fleiss’ K*	0.08; 0.42	fleiss.kappa.raw: 0.07; 0.41
	Conger’s K*	0.08; 0.41	conger.kappa.raw: 0.09; 0.41
	BP*	0.69; 0.87	bp.coeff.raw: 0.69; 0.86
	S*	0.69; 0.85	wlin.conc: 0.70; 0.85
	Hubert’s K*	0.08; 0.41	N.A.
**AP3** **(n = 46)**	*α*	−0.09; 0.20	krippen.alpha.raw: −0.11; 0.20
	Fleiss’ *K*	−0.11; 0.20	fleiss.kappa.raw: −0.12; 0.19
	Light’s *K*	−0.09; 0.17	N.A.
	Hubert’s *K*	−0.12; 0.16	N.A.
	Conger’s *K*	−0.10; 0.18	conger.kappa.raw: −0.11; 0.19
	*BP* coefficient	0.56; 0.83	bp.coeff.raw: 0.56; 0.83
	*S*	0.57; 0.82	concordance: 0.57; 0.83
	*γ*(*AC*_1_)	0.66; 0.88	gwet.ac1.raw: 0.66; 0.89
	Δ	0.09; 0.86	N.A.
	α*	−0.07; 0.23	krippen.alpha.raw: −0.09; 0.23
	*γ*(*AC*_2_)	0.81; 0.95	gwet.ac1.raw: 0.81; 0.94
	Fleiss’ K*	−0.08; 0.22	fleiss.kappa.raw: −0.09; 0.22
	Conger’s K*	−0.07; 0.22	conger.kappa.raw: −0.08; 0.22
	BP*	0.67; 0.88	bp.coeff.raw: 0.67; 0.88
	S*	0.68; 0.87	wlin.conc: 0.66; 0.87
	Hubert’s K*	−0.07; 0.21	N.A.

Abbreviations: n = sample size; *α* = Krippendorff’s *α*; *S* = Quatto’s *S*; *γ*(*AC*_1_) = Gwet’s *γ*(*AC*_1_); Δ = Andrés and Hernàndez’s multi-raters Δ; α* = Krippendorff’s weighted *α*; *γ*(*AC*_2_) = Gwet’s *γ*(*AC*_2_); Fleiss’ K* = Fleiss’ weighted *K*; Conger’s K* = Conger’s weighted *K*; BP* = Brennan and Prediger’s weighted *BP* coefficient; S* = Quatto’s weighted *S*; Hubert’s K* = Hubert’s weighted *K*; AP1 = alpine pasture 1; AP2 = alpine pasture 2; AP3 = alpine pasture 3; N.A. = not available (i.e., no R function available to calculate confidence intervals).

## Data Availability

The raw data supporting the conclusions of this article will be made available by the authors on request.

## Supplementary Materials

The following supporting information can be downloaded at: https://www.mdpi.com/article/10.3390/ani16040546/s1, Supplementary Materials: Agreement indices.

## Abbreviations

The following abbreviations are used in this manuscript:

IOR	Inter-observer reliability
UA	Udder asymmetry
BCS	Body condition score
AP	Alpine pasture
