# Supplementary material for: Comparing Agreement Indices to Assess Inter-Observer Reliability in the Case of Dichotomous and Trichotomous Animal-Based Welfare Indicators with Three Raters
Source: Animals (Basel). 2026 Feb 10;16(4):546. doi: 10.3390/ani16040546 (PMC12937283; doi:10.3390/ani16040546)
Supplement: Supplementary file 1 [file animals-16-00546-s001.zip › animals-4125018-supplementary.pdf]

Supplementary Materials - Agreement indices

The closed formulas of the considered agreement indices are presented below, following the chronological order of their development. The general structure of all these indices is the same. Some agreement indices share the same concordance rate (i.e., percentage of agreement; P_0); the concordance rate can differ from one index to the other depending on the exploited concordance matrix. The agreement indices mainly differ each other for the chance agreement (P_e). The performance of the indices varies based on the different calculation of P_e, whose formulas are given below. The best agreement indices are those ones able to confer the lowest P_e.

The general formula implemented for each agreement index is the following one:

$$General\ formula\ for\ the\ agreement\ indices = (P_0 - Pe)/(1 - Pe) \quad (S1)$$

In (S1), P_0 is the observed agreement, while P_e is the agreement which occurs by chance.

Based on the exploited concordant matrix, three different values of P_0 are obtained, and the formulas proposed in the literature for such implementation are the following ones:

$$P_0 = \frac{1}{n'} \sum_{i=1}^{n'} \sum_{k=1}^{q} \frac{r_{ik}(r_{ik} - 1)}{r_i(r_i - 1)} \quad (S2)$$

This P_0 (S2) is used in the case of the unweighted indices belonging to the Kappa statistic, with few exceptions (i.e., Hubert's *K*) and in the case of Brennan and Prediger's (*BP*) coefficient, Quatto's *S* and Gwet's $\gamma(AC_1)$. In (S2), r_i represents the total number of raters who score a subject *i*, while r_{ik} represents the total number of raters who classify the subject *i* within the category *k* [11].

For Krippendorff's α another type of formula was proposed, even if such formula derives from the same concordance matrix developed for the above-mentioned indices, and which consequently conduces to the same value of P_0:

$$P_0' = (1 - \varepsilon_n)P_0 + \varepsilon_n \quad (S3)$$

In this case, P_0 is the value obtained from (S2), while ε_n is equal to:

$$\varepsilon_n = 1/n'\bar{r} \quad (S4)$$

In (S4) n' is the number of subjects classified by the raters, while $\bar{r}$ is the average number of raters who classify a subject [11].

Concerning Hubert's *K* and Andrès and Hernàndez's multi-raters *Δ*, another value of P_0 is obtained, as the concordant matrix proposed for such implementation is different. In this case, the general formula proposed for the implementation of P_0 is the following one:

$$\hat{P}_0 = \sum_{i=1}^{K} \hat{p}_i \quad \text{(S5)}$$

In (S5) $\hat{p}_i$ is the estimated proportion of agreement in the category *i* [23].

Concerning the weighted agreement indices, another value of P_0 is obtained, depending on the concordance matrix developed for their implementation. A formula of P_0, common to the most of the implemented weighted agreement indices (with few exceptions, such as Krippendorff's weighted α and Hubert's weighted *K*), is given by Gwet [11]:

$$P_0 = \frac{1}{n'} \sum_{i=1}^{n'} \sum_{k=1}^{q} \frac{r_{ik}(r_{ik}^{*} - 1)}{r_i(r_i - 1)} \quad \text{(S6)}$$

where r_{ik} is the total number of raters who classify the subject *i* within the *k* category, while r_i is the total number of raters who score the subject *i* [11]. Such formula (S6) is identical to the formula (S2), but it differs in the use of the weights for the implementation of P_0.

Gwet [11] and Andrès and Hernàndez [30] also proposed a new implementation of P_0 for Krippendorff's weighted α and Hubert's weighted *K*. These two formulas are different from (S6), but they are developed from the same concordance matrix, consequently conducing to the same result:

$$P_0' = \frac{1}{n'} \sum_{i=1}^{n'} \sum_{k=1}^{q} \frac{r_{ik}(r_{ik}^{*} - 1)}{\bar{r}(r_i - 1)} \quad \text{(S7)}$$

Gwet [11] proposed (S7) for the implementation of P_0 for Krippendorff's weighted α, where n' is the number of subjects evaluated by the raters, r_i is the total number of raters who score the subject i and $\bar{r}$ is the average number of raters who classify a subject.

On the other hand, the formula proposed by Andrès and Hernàndez [30] in the case of Hubert's weighted K is given below:

$$P_0 = \sum_{r} \sum_{r' \neq r} \frac{P_0(r, r')}{\{R(R-1)\}} \qquad \text{(S8)}$$

In (S8), the pairwise agreement for every pair of raters involved in the evaluation (r, r') is considered, as well as the effective total number of raters involved.

S.1. Krippendorff's α

Krippendorff's α [13] is implemented to assess the agreement between two or more raters and in the presence of categorical variables, characterised by any number of categories.

According to Gwet [11] the P_e for α is given by:

$$P_e = \sum_{k=1}^{q} \hat{\pi}_k^2 \qquad \text{(S9)}$$

where $\hat{\pi}_k$ is defined as the probability for the raters to casually classify a subject within a preselected category:

$$\hat{\pi}_k = \frac{1}{n} \sum_{i=1}^{n} \frac{r_{ik}}{r_i} \qquad \text{(S10)}$$

Krippendorff's α values can vary from 0 (absence of agreement among raters) to 1 (perfect agreement among raters) [13].

S.2. Fleiss' K

Fleiss' K [12] and Krippendorff's α are very close each other.

Fleiss' K is used to assess the agreement among many raters in the case of categorical variables [12]. This index is a generalisation of Scott's π [38] when the evaluations are made in the presence of more than two raters.

For the implementation of the chance agreement (P_e), Fleiss [12] starts from the assumption of the independence among the raters when they randomly classify a subject within a preselected category, thus without influencing each other [11]. Consequently, the probability of this casual classification is always given by $\hat{\pi}_k$, which has been previously defined (S10).

Then, starting from (S10), the P_e is defined as the probability that a pair of raters assign a subject within the same category:

$$P_e = \sum_{k=1}^{q} \hat{\pi}_k^2 \qquad (S11)$$

When Fleiss' K is equal to 1, the raters completely agree, while when Fleiss' K is equal or lower than 0, the raters do not agree.

S.3. Light's *K*

Light [15] generalised the Kappa statistic when the evaluations are made in the presence of more than two raters, which are able to assign a certain categorical variable within a pre-established category. Light averaged the agreement obtained for each pair of raters implementing Cohen's K [21], thus obtaining Light's K index. Consequently, the calculation of the P_e for Light's K follows the formula proposed for Cohen's K [11]:

$$P_e = \sum_{k=1}^{q} p_{k+} p_{+k} \qquad (S12)$$

where p_{k+} represents the percentage of subjects which are assigned by rater A to the category k, while p_{+k} represents the percentage of subject which are given by rater B to the same category [11].

S.4. Hubert's *K*

Hubert [16] extended the Kappa statistic in the case of evaluations performed by more than two raters for categorical variables. Specifically, for Hubert the concordance occurs when all the raters agree in assigning a variable inside a certain category [23]. The general formula proposed for the implementation of the P_e is presented below:

$$P_e = \sum\nolimits_{i=1}^{K} P_i \qquad (S13)$$

where P_i is the level of concordance present among the raters when a subject is classified within a predetermined category. P_i is implemented through the following formula:

$$P_i = \prod_{r=1}^{R} t_{ir} \quad (S14)$$

where t_{ir} represents the total number of subjects classified within the category *i* by the rater *r* [23].

S.5. Conger's *K*

Conger [14] extended the Kappa statistic formulated by Fleiss [12], which considers the pairwise agreement among raters, to a more generalised statistic, which is able to define a total agreement among *n* raters, in the presence of categorical variables. In particular, the overall formula for the Kappa statistic proposed by Conger is able to consider not only the pairs of raters which agree, but also the total agreement among all the raters involved in the evaluation. The approach proposed by Conger is different if compared to the one proposed by Light [15]. Indeed, the P_e proposed by Conger [14] is obtained averaging the chance agreement of all the pairs of raters involved in the evaluation process [11]. However, averaging all pairwise chance agreement could result in a labour process if the number of raters is higher than three. At this regard, Conger [14] developed an alternative formula which can allow to obtain a direct calculation of the P_e:

$$P_e = \sum_{k=1}^{q} \bar{p}^2{\cdot}_k - \sum_{k=1}^{q} s^2{}_k/r \quad (S15)$$

where $s^2{}_k$, which is the sample variance of the *r* proportion $p_{1k}\ldots, p_{rk}$, is given by the following formula:

$$s^2{}_k = \frac{1}{r-1}\sum_{g=1}^{r}\left(p_{gk} - \bar{p}_k\right)^2 \quad (S16)$$

In (S16) p_{gk} is the proportion of individuals classified by the rater *g* within the category *k*, while $\bar{p}_k$ is the average value of the probabilities ($p_{1k}\ldots, p_{rk}$) [11].

S.6. Quatto's *S*

Quatto's *S* [25] is implemented to assess the agreement among two or more raters, and in the presence of categorical variables characterised by any number of categories.

The P_e obtained for Quatto's *S* is calculated as:

$$P_e = 1/M \quad (S17)$$

where *M* represents the total number of categories characterising the variable. A similar implementation of P_e is given in Bennett et al. [51] and Brennan and Prediger [24].

S.7. Krippendorff's weighted α

Krippendorff's weighted α [29] is the weighted form of Krippendorff's α [13], and it is mainly exploited for assessing IOR for ordinal variables and in the presence of two and multiple raters.

The P_e for such index is given by [11]:

$$P_e = \sum_{k,l} w_{kl} \pi_k \pi_l \quad \text{(S18)}$$

In (S18) w_{kl} represents the exploited weights.

S.8. Gwet's $\gamma(AC_1)$

Gwet's $\gamma(AC_1)$ [27] is implemented to assess the agreement among two or more raters and in the presence of categorical variables characterised by any number of categories.

As observed for Quatto's *S* [25], also Gwet's $\gamma(AC_1)$ considers the number of categories during the implementation of the chance agreement, which results challenging if compared to the implementation of the chance agreement for the *S*-index. Specifically, Gwet [27] proposed the following formula for the calculation of the P_e:

$$P_e = \frac{1}{q(q-1)} \sum_{k=1}^{q} \hat{\pi}_k (1 - \hat{\pi}_k) \quad \text{(S19)}$$

In (S19) q represents the total number of categories characterising the variable under analysis.

Gwet's $\gamma(AC_1)$ can assume agreement values ranging from 0 (absence of agreement among the raters) to 1 (presence of total agreement among the raters).

S.9. Gwet's $\gamma(AC_2)$

Gwet's $\gamma(AC_2)$ [11] is the weighted form of the $\gamma(AC_1)$ index [27]. This index is used to evaluate the agreement among two or more raters, and in the presence of ordinal variables characterised by any number of categories.

The formula for the calculation of the P_e proposed by Gwet [11] is the following one:

$$P_e = \frac{T_w}{q(q-1)} \sum_{k=1}^{q} \pi_k (1 - \pi_k) \quad \text{(S20)}$$

where q represents the number of categories which characterises the variable under analysis, T_w is given by the sum of all the considered weights, and π_k is the propensity to assign a subject within a specific category.

S.10. Fleiss' weighted *K*

Fleiss' weighted *K* [11] is the weighted version of the generalised Fleiss' *K* [12], and it is implemented to assess the IOR for ordinal variables and in the presence of three or more raters. The formula proposed by Gwet [11] for the calculation of P_e is given by:

$$P_e = \sum_{k,l} w_{kl} \pi_k \, \pi_l \quad (S21)$$

In (S21) w_{kl} represents the relative weights.

S.11. Conger's weighted *K*

Conger's weighted *K* [11] is the weighted version of the generalised Conger's *K* [14]. It is developed to assess the IOR for ordinal variables, in the presence of three raters or more. The formula proposed by Gwet [11] for the implementation of P_e is given by:

$$P_e = \sum_{k,l} w_{kl} (\bar{p} \, + k\bar{p} + l - \, s_{kl}^2 / r) \quad (S22)$$

where w_{kl} represents the relative weights.

S.12. Quatto's weighted *S*

Quatto's weighted *S* [26] is the weighted form of Quatto's *S* [25]. This index is implemented to assess the agreement among two or more raters, and in the presence of ordinal variables characterised by any number of categories.

The P_e for Quatto's weighted *S* is given by Marasini et al. [26]:

$$P_e = \frac{1}{M} + \frac{2}{M^2} \sum_{j=1}^{M-1} \sum_{k>j}^{M} w_{jk} \quad (S23)$$

where *M* represents the total number of categories characterising the variable under analysis.

A similar implementation of the P_e is given by Gwet [11] for the Brennan and Prediger's (*BP*) coefficient.

In the case of linear weights [52] proposed by Marasini et al. [26] for this kind of index, the latter formula (S23) simplifies as follows:

$$P_e = \, (2M - 1)/3M \quad (S24)$$

S.13. Andrès and Hernàndez's multi-raters *Δ*

Andrès and Hernàndez's multi-raters Δ [28] is an extension of Andrès and Marzo's Δ [49] for assessing the agreement among more than two raters, and in the presence of categorical variables. Multi-raters Δ can be expressed using the following formula:

$$multi - raters\ \Delta = \sum_{i=1}^{k} \hat{a}_i \quad (S25)$$

where $\hat{a}_i$ represents the estimated level of agreement between the raters when they classify a subject within a predetermined category i [28].

However, an easier method to implement this index is given by the traditional formula (S1).

In the latter case, the calculation of P_e for multi-raters Δ is given by:

$$P_e = \sum_{i=1}^{K} \prod_{r=1}^{R} \pi_{ir} \quad (S26)$$

where π_{ir} represents the total probabilities of assignments of each subject to a predetermined category [28].

S.14. Hubert's weighted *K*

Hubert's weighted *K* [30] is the weighted version of the generalised Hubert's *K* [16]. This index is implemented to assess the IOR of ordinal variables, and in the presence of three raters or more.

The P_e calculated for Hubert's weighted *K* follows the pairwise method proposed by Fleiss [12], for which each pair of raters agree in assigning a subject within the same category [30]. The formula proposed by Andrès and Hernàndez [30] is the following one:

$$P_e = \sum_i \sum_j w_{ij} \left(p_i + p_{j+} - \sum_r p_{ir} p_{jr} \right) / \{R(R-1)\} \quad (S27)$$

In (S27) R represents the number of raters involved in the evaluation, while w_{ij} represents the relative weights.
